# Cannabinoid Receptor Signaling in Central Regulation of Feeding Behavior: A Mini-Review

**DOI:** 10.3389/fnins.2017.00293

**Published:** 2017-05-24

**Authors:** Marco Koch

**Affiliations:** Medical Faculty, Institute of Anatomy, University of LeipzigLeipzig, Germany

**Keywords:** cannabinoid receptor type 1, endocannabinoids, hypothalamus, feeding behavior, anorexia, cachexia, overeating, obesity

## Abstract

Cannabinoids are lipid messengers that modulate a variety of physiological processes and modify the generation of specific behaviors. In this regard, the cannabinoid receptor type 1 (CB_1_) represents the most relevant target molecule of cannabinoids so far. One main function of central CB_1_ signaling is to maintain whole body energy homeostasis. Thus, cannabinoids functionally interact with classical neurotransmitters in neural networks that control energy metabolism and feeding behavior. The promotion of CB_1_ signaling can increase appetite and stimulate feeding, while blockade of CB_1_ suppresses hunger and induces hypophagia. However, in order to treat overeating, pharmacological blockade of CB_1_ by the inverse agonist rimonabant not only suppressed feeding but also resulted in psychiatric side effects. Therefore, research within the last decade focused on deciphering the underlying cellular and molecular mechanisms of central cannabinoid signaling that control feeding and other behaviors, with the overall aim still being the identification of specific targets to develop safe pharmacological interventions for the treatment of obesity. Today, many studies unraveled the subcellular localization of CB_1_ and the function of cannabinoids in neurons and glial cells within circumscribed brain regions that represent integral parts of neural circuitries controlling feeding behavior. Here, these novel experimental findings will be summarized and recent advances in understanding the mechanisms of CB_1_-dependent cannabinoid signaling being relevant for central regulation of feeding behavior will be highlighted. Finally, presumed alternative pathways of cannabinoids that are not driven by CB_1_ activation but also contributing to control of feeding behavior will be introduced.

## Introduction

Central regulation of feeding behavior is indispensable to life, since animals and men have to consume energy in terms of food to exert essential daily functions (Gao and Horvath, 2016). In this regard, a network of neural circuitries evolved that ensures constant energy supply by providing a “pro-feeding” behavioral outcome: in times when food is plentiful, energy intake dominates energy expenditure, so that excessive energy could be stored and used when food was restricted or temporarily not available (Koch and Horvath, 2014).

Cannabinoids, such as THC interfere with central regulation of feeding behavior by acting upon G protein-coupled cannabinoid receptor type 1 (CB_1_) in the brain (Williams and Kirkham, 1999). However, the underlying molecular and cellular mechanisms of central CB_1_ signaling in control of feeding and other behaviors are still far from being fully understood (Mazier et al., 2015). Moreover, better insight into the aforementioned network being responsible for central control of feeding behavior is of significant interest, since nowadays, the respective neural circuitries are of substantial clinical relevance. Most importantly, availability of food no longer represents an evolutionary pressure, since food exists in abundance in many (albeit not all) countries around the world. Moreover, energy-dense foods high in carbohydrates and rich in fat can be obtained with little or no efforts. Thus, many people are suffering from chronic overload with nutrients in today's world, which, when accompanied by overall decreased physical activity is often leading to a morbid increase in body fat mass and resulting in obesity. On the other hand, a significant number of patients is affected from a complete loss of appetite (anorexia), which may be caused by psychiatric disorders, or by cancer and infectious diseases, and make these patients suffering from chronic under-nutrition (Scarlett and Marks, 2005; Park et al., 2014). Thus, decoding of the underlying cellular and molecular mechanisms in the central nervous system (CNS) that control feeding behavior may help to develop pharmacological interventions not only for disorders related with anorexia, but also for the treatment of the ever-increasing number of obese patients worldwide (Dietrich and Horvath, 2012).

Since time immemorial, cannabis extracts are used for recreational purposes. However, it is clear today that not only the psychotropic properties but also the well-known appetite stimulating effects of the plant-derived cannabinoid THC are mediated by CB_1_ activation (Silvestri and Di Marzo, 2013). CB_1_ belongs to the endocannabinoid system (ECS) that further consists of endocannabinoids (eCBs) as intrinsic CB_1_ ligands, and of eCB synthesizing and hydrolyzing enzymes (Piomelli, 2003). These enzymes steadily control eCB levels in a temporal and spatial fashion to guaranty functional CB_1_ signaling in a region and cell type specific manner (Pertwee, 2014). Interestingly, malfunction of the central ECS is associated with overeating and obesity (Engeli, 2008; Mazier et al., 2015). Thus, the main purpose here is to summarize recent experimental findings for central control of feeding behavior in health and disease, with special focus on central CB_1_ signaling. Finally, presumed alternative, non-CB_1_ driven pathways by which eCBs might also contribute to feeding regulation will be introduced.

## Does CB_1_ still lend itself as a therapeutic target in central feeding regulation?

CB_1_ was discovered almost 30 years ago and later identified as a promising target molecule in the CNS to pharmacologically interfere with feeding behavior (Matsuda et al., 1990; Devane et al., 1992; Williams and Kirkham, 1999). Besides feeding, several other physiological functions, and behaviors being modulated by central CB_1_ signaling were deciphered so far (Lutz et al., 2015), and many pharmacological, biochemical, and morphological aspects of central CB_1_ signaling were characterized.

**Figure 1:**
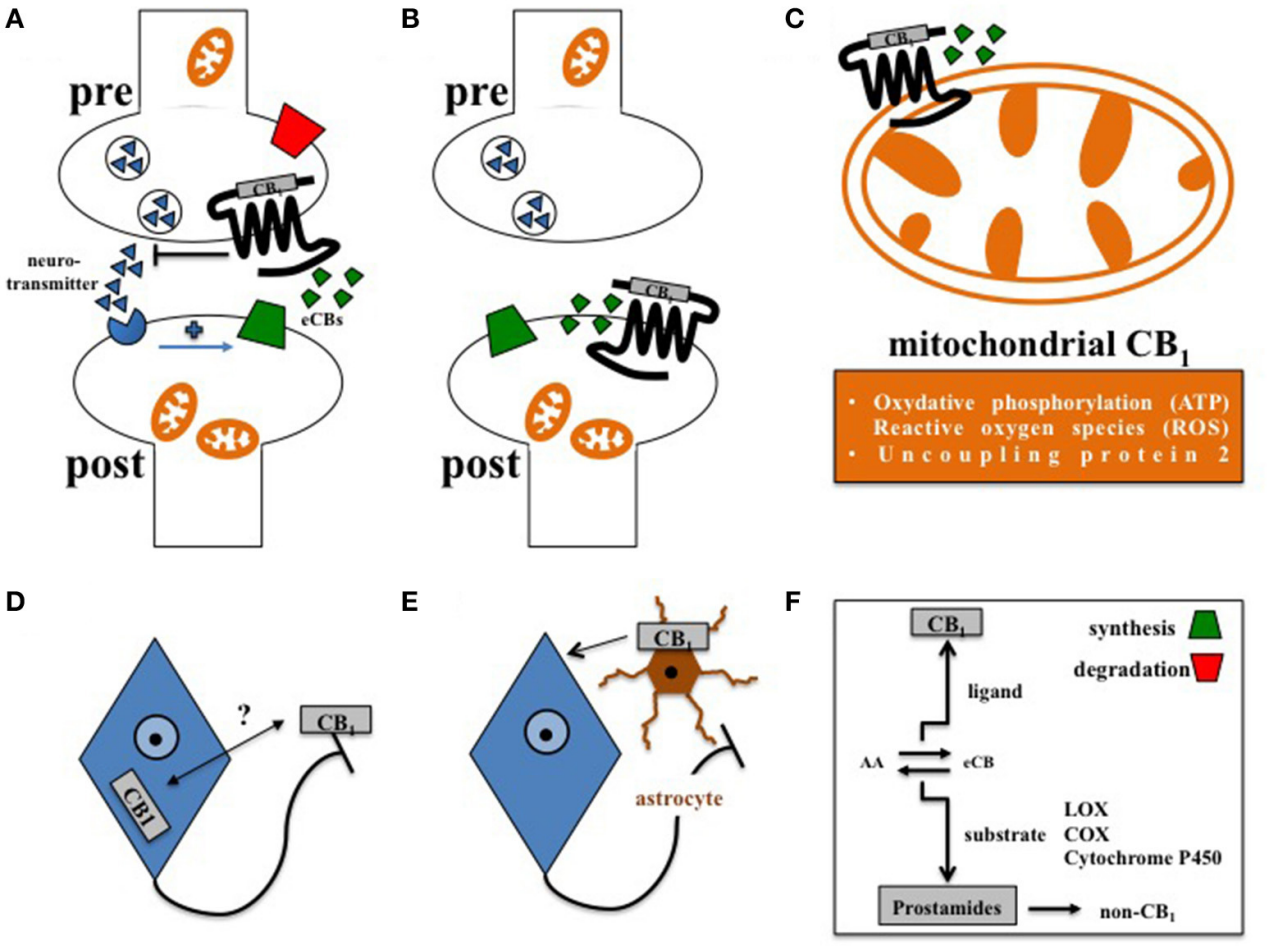
**Principles of central CB_**1**_ signaling in control of feeding behavior. (A)** Retrograde signaling of eCBs at presynaptic CB_1_ impacts feeding (Bellocchio et al., 2010). **(B)** Postsynaptic CB_1_ at POMC neurons affects feeding in DIO (Morello et al., 2016). **(C)** Cannabinoids interfere with mitochondrial CB_1_ in hypothalamic feeding regulation (Koch et al., 2015). **(D)** Whether activity-dependent subcellular distribution of CB_1_(Thibault et al., 2013) accounts for control of food intake is still open. **(E)** Astroglial CB_1_ regulates the metabolic effects of leptin in cultured astrocytes (Bosier et al., 2013), and thus might contribute to astrocyte-dependent control of feeding behavior in the hypothalamus (Kim et al., 2014). **(F)** Enzymes of eCB synthesis or degradation control eCB levels in a spatial and temporal manner (Pertwee, 2014). Moreover, eCBs not only function as CB_1_ ligands, but also as substrates of specific enzymes, such as lipoxygenases (LOX), cyclooxygenases (COX), or cytochrome P450, supporting the idea that the ECS might also transmit metabolic effects independently from CB_1_ signaling (non-CB_1_).

The vast majority of CB_1_ is located at presynaptic terminals in order to suppress the further release of classical neurotransmitters, such as GABA or glutamate (Castillo et al., 2012). However, different localizations and functions of CB_1_ were also discovered (Figure 1). In principle, the acute pharmacological promotion of central CB_1_ signaling can evoke food intake and thus still represents a promising approach to treat anorexia (Williams and Kirkham, 1999; Aigner et al., 2011; Reuter and Martin, 2016). However, it was discovered a couple of years ago that only administration of low to moderate doses of CB_1_ agonists were able to increase food intake in mice, while moderate to high doses of CB_1_ agonists decreased feeding (Bellocchio et al., 2010). In this, hypophagia was induced by CB_1_-mediated reduction of GABAergic transmission, while hyperphagia was stimulated by CB_1_-driven suppression of glutamatergic conduction (Bellocchio et al., 2010; Busquets Garcia et al., 2016). This fundamental finding in mice might explain the contrary results of different clinical trials on the use of CB_1_ agonists in order to treat anorexia in humans (Aigner et al., 2011; Reuter and Martin, 2016). Thus, further approaches are needed to carefully reconsider the beneficial effects of CB_1_ agonists for the treatment of anorexia (Whiting et al., 2015). In contrast to CB_1_ agonists, the overall blockade of CB_1_ by rimonabant generally suppressed hunger and induced hypophagia (Colombo et al., 1998; Simiand et al., 1998), but unfortunately also resulted in psychiatric side effects in humans. To develop more specific and safe pharmacological interventions for the treatment of overeating, the recently presented molecular ultrastructure of human CB_1_ may deliver new opportunities for the design of next-generation CB_1_ directing pharmaceuticals as novel anti-obesity drugs (Hua et al., 2016; Shao et al., 2016). Moreover, allosteric agents directed against CB_1_ such as hemopressin or pregnenolone (Heimann et al., 2007; Dodd et al., 2010, 2013; Vallee et al., 2014) may supply medications with a significantly improved side effect profile (Busquets Garcia et al., 2016). Finally, another pharmacological approach aimed at selective blockade of peripheral CB_1_, which basically was shown to induce metabolic benefits independently from modification of feeding behavior (Nogueiras et al., 2008; Tam et al., 2012). Nevertheless, it is primarily the knowledge about the cell type specific functions of CB_1_ signaling in different types of neurons, and, as discussed later, also in glial cells, such as astrocytes (Metna-Laurent and Marsicano, 2015), which will determine if and in how far the full therapeutic potential of CB_1_ pharmacology in feeding regulation can be leveraged.

In this regard, complexity of central CB_1_ signaling was further broaden by the observation that CB_1_, as a G protein-coupled receptor, is not exclusively expressed at the plasma membrane but also located at the outer mitochondrial membrane (Benard et al., 2012; Hebert-Chatelain et al., 2014). By interfering with respiratory chain complex I, mitochondrial CB_1_ was recently shown to promote the amnesia-inducing effects of CB_1_ agonists in the hippocampus (Hebert-Chatelain et al., 2016; Harkany and Horvath, 2017). Accordingly, effects of cannabinoids on food intake are also transmitted via CB_1_-induced mitochondrial adaptations, since induction of feeding by CB_1_ agonists depended on the expression of mitochondrial uncoupling protein 2 and the formation of reactive oxygen species (ROS) in the hypothalamus (Koch et al., 2015; Kruger, 2016), finally pointing toward region-specific functions of mitochondrial CB_1_ signaling in the brain (Harkany and Horvath, 2017). However, CB_1_ driven control of ROS seems to be multifaceted, since cannabinoids reduced leptin-mediated ROS formation in cultured hypothalamic neurons by CB_1_ dependent peroxisome proliferator-activated receptors (PPAR)-gamma and subsequent catalase activation (Palomba et al., 2015). Overall, about 15% of total brain CB_1_ is associated with mitochondria (Benard et al., 2012; Hebert-Chatelain et al., 2014), and it appeared that CB_1_ is present in mitochondria of both pre- and postsynaptic terminals (Busquets Garcia et al., 2016). However, CB_1_ is most abundantly expressed at the plasma membrane of axonal shafts and presynaptic terminals (Pertwee, 2010), and significant amounts of CB_1_ in the forebrain are constantly activated, internalized, and recycled at steady state (Thibault et al., 2013). Whether internalization and redistribution of CB_1_ between axonal plasma membrane and somato-dendritic endosomes account for control of feeding behavior still needs to be investigated. Moreover, functional expression of CB_1_ is also observed at the postsynaptic plasma membrane (Castillo et al., 2012). In the course of diet-induced obesity (DIO), orexin-A represses satiety-promoting pro-opiomelanocortin (POMC) neurons in the hypothalamic arcuate nucleus (ARC) by eCB-mediated activation of postsynaptic CB_1_ on POMC neurons (Morello et al., 2016).

In addition to neurons, CB_1_ is also expressed in astrocytes (Metna-Laurent and Marsicano, 2015; Oliveira Da Cruz et al., 2016), and plays an important role in neuroinflammation (Walter and Stella, 2004), and in physiological neurotransmission (Navarrete and Araque, 2010; Han et al., 2012). Interestingly, astrocyte-dependent energetic support of neurons also involves CB_1_, since leptin-induced astroglial glycogen accumulation depends on CB_1_ signaling in cultured astrocytes (Bosier et al., 2013). However, the relevance of astroglial CB_1_ in distinct hypothalamic feeding centers has to be considered *in vivo*. Accordingly, structural analyses determined CB_1_ in the immediate vicinity to astrocytes at tripartite synapses in the ARC (Morozov et al., 2017). Moreover, hypothalamic astrocytes and microglia show morphological adaptations in DIO (Baufeld et al., 2016; Argente-Arizon et al., 2017), and astrocytes, via leptin signaling, actively control hypothalamic neuronal circuits, and feeding (Kim et al., 2014). Thus, it is of significant interest to study the function of CB_1_ signaling in glial cells under normal and high fat diet (HFD).

Together, studies focusing on the cell type specific expression and subcellular distribution of CB_1_ delivered unique mechanistic insights into central CB_1_ signaling, which provides an important prerequisite to uncover the physiological role of CB_1_ in distinct homeostatic and hedonic feeding centers of the CNS.

## Recent advances in understanding homeostatic and hedonic feeding control: what is the relevance of CB_1_?

Homeostatic feeding centers supervise the body's energy resources and are located in the hypothalamus and caudal brainstem (Koch and Horvath, 2014), while hedonic feeding centers relevant for palatability and rewarding aspects of food are pinpointed to the mesolimbic system (Alonso-Alonso et al., 2015; Pandurangan and Hwang, 2015). Although both control systems are anatomically located in different brain areas, it becomes more likely that they are functionally closely interconnected to each other (Munzberg et al., 2016).

CB_1_ obtains a conserved distribution in the CNS among different mammalian species (Herkenham et al., 1990). High CB_1_ expression levels in the hippocampus or basal ganglia are attributed to cannabinoid-induced effects on memory formation and movement (Castillo et al., 2012). Low CB_1_ expression levels in hypothalamic or caudal brainstem nuclei display significant functions in regulation of feeding behavior (Cardinal et al., 2012; Mazier et al., 2015). In this, distinct groups of hypothalamic neurons measure the body's energy resources by sensing circulating nutrients and detecting metabolic hormones, such as leptin, insulin, or ghrelin (Varela and Horvath, 2012; Vogt and Bruning, 2013; Muller et al., 2015). Moreover, hypothalamic neurons are directly affected by cannabinoids, since infusion of CB_1_ agonists into distinct hypothalamic nuclei acutely induced feeding (Jamshidi and Taylor, 2001; Koch et al., 2015). Interestingly, hypothalamic CB_1_ signaling interferes with signal transmission of metabolic hormones. While leptin suppressed feeding correlates with decreased hypothalamic eCB levels (Di Marzo et al., 2001), ghrelin triggered acute feeding accompanies with increased hypothalamic eCB levels, and depends on paraventricular nucleus (PVN) CB_1_ signaling (Kola et al., 2008). However, CB_1_ mediated control of feeding in the PVN is more complex than thought before, since under an experimental fasting/re-feeding paradigm, blockade of local CB_1_ in the PVN increased hyperphagy in hungry mice, and enhanced the hyperphagic effect of ghrelin in fed animals (Soria-Gomez et al., 2014b). Thus, hypothalamic eCBs represent local neuromodulators that are actively involved in rapid rewiring of hypothalamic feeding circuits in accordance to the current prandial state (Pinto et al., 2004). In DIO, imbalanced hypothalamic eCB levels and defective CB_1_ signaling seem to be the consequence of central leptin resistance (Silvestri and Di Marzo, 2013). In the lateral hypothalamus (LH), CB_1_ is involved in physiological control of melanin-concentrating hormone and orexin-A neurons (Silvestri and Di Marzo, 2013). In DIO, eCBs in the LH promote hyperphagia by remodeling the synaptic input organization of orexin-A neurons (Alpar and Harkany, 2013; Cristino et al., 2013).

In the ARC, at least two neuronal populations with opposing effects on feeding behavior can be distinguished: the hunger promoting Agouti-related protein/neuropeptide Y (AgRP/NPY) neurons that acutely promote food intake, and POMC neurons that drive gradual onset of satiety (Varela and Horvath, 2012). Systemic blockade of CB_1_ by rimonabant reduced NPY levels, indicating that AgRP/NPY neurons are controlled by local eCBs (Verty et al., 2009). AgRP/NPY neurons do not contain CB_1_ (Cota et al., 2003; Horvath, 2003), but CB_1_ was predominately found at GABAergic terminals innervating AgRP/NPY neurons (Morozov et al., 2017). Thus, local eCBs in the ARC might promote feeding by retrograde dis-inhibition of AgRP/NPY neurons. However, POMC neurons are also affected by cannabinoids via pre- and postsynaptic CB_1_ (Hentges et al., 2005; Koch et al., 2015; Morello et al., 2016). In fed mice, CB_1_ agonists rapidly converted POMC neurons from promoters of long-term satiety into acute drivers of hunger (Koch et al., 2015; Patel and Cone, 2015). In DIO, orexin-A repressed POMC neurons by constitutive eCB signaling at postsynaptic CB_1_ in POMC neurons (Morello et al., 2016). Mapping of hypothalamic neuronal subtypes by single-cell RNA sequencing (Romanov et al., 2017) and molecular indexing of local ARC cell types by gene expression profiling identified novel cell types of putative relevance for regulation of distinct vegetative body functions, including feeding (Campbell et al., 2017). Thus, it would be interesting to dissect the functional relevance of CB_1_ signaling in these cell types. Accordingly, glutamate-releasing neurons in the ARC that express oxytocin receptors were identified as an integral part of a rapid ARC to PVN satiety pathway (Fenselau et al., 2017). However, whether acute effects of cannabinoids on feeding might be further transmitted by this novel pathway remains elusive. Alongside, local ARC dopaminergic cells were identified that reciprocally control activity of AgRP/NPY and POMC neurons (Zhang and Van Den Pol, 2016). This finding is of substantial interest in order to study CB_1_ controlled homeostatic feeding, since dopamine modulates rewarding aspects of food mainly through dopaminergic ventral tegmental area (VTA) to nucleus accumbens (NAc) projections (Volkow et al., 2011), and CB_1_ signaling was shown to modulate dopaminergic signaling in the NAc and VTA to regulate hedonic aspects of feeding (Melis et al., 2007; Di Marzo et al., 2009).

Beside the VTA located in the rostral brainstem, CB_1_ signaling is also interfering with the functional activity of caudal brainstem nuclei, such as parabrachial nucleus, dorsal motor nucleus of the vagus, and nucleus of the solitary tract. In this, CB_1_ basically controls food preferences, such as digestion of palatable foods being rich in fat (Busquets Garcia et al., 2016). Finally, hypothalamic AgRP/NPY and POMC neurons are not only directly affected by food intake itself, but also rapidly respond to sensory detection of available food (Chen et al., 2015). It is thus likely that hypothalamic neurons not only transmit internal signals causing hunger or satiety in response to eating and internal sensing of energy resources, but also receive external information on the incentive value of food, such as sight, smell, and taste in order to rapidly react to food stimuli and transmit motivational aspects on feeding being generated via the mesolimbic system (Seeley and Berridge, 2015). Processing of food sensations such as olfactory or gustatory signals indeed involve CB_1_ signaling, since fasted mice displayed CB_1_-dependent increased odor detection in the main olfactory bulb (Soria-Gomez et al., 2014a).

## Besides CB_1_: does the ECS provide other relevant target molecules in feeding regulation?

Within the ECS, it is the availability of eCBs that provides the routes and directions of CB_1_ signaling in the brain. While research was long-time focusing on pharmacological modulation of CB_1_ signaling by direct interaction at CB_1_ in order to interfere with feeding and other behaviors, numerous evidence arose that targeting of classical enzymes involved in biosynthesis or degradation of eCBs will also allow to induce adaptations in feeding behaviors (Pertwee, 2014). For example, degradation of the eCB 2-arachidonoylglycerol (2-AG) into arachidonic acid and glycerol is basically controlled by three different serine hydrolases: while monoacylglycerol lipase (MAGL) accounts for 85% of 2-AG degradation, alpha/beta-hydrolase domain containing (ABHD) 6, and 12 are responsible for hydrolysis of 5 and 10%, respectively (Savinainen et al., 2012). Indeed, it was shown that knockdown of ABHD6 in the ventromedial hypothalamus resulted in locally elevated 2-AG levels, finally resulting in a blunted fasting-induced feeding response and in a general diminished efficacy of the mice in order to adapt to other metabolic shifts (Fisette et al., 2016).

Generally, eCBs do not resemble to classical neurotransmitters that are stored in synaptic vesicles (Piomelli, 2003). Instead, eCBs, as being arachidonic acid derivatives, are produced on demand from lipid precursors. Most eCBs display a relative short half-life, since they are attracted by both classical eCB degrading enzymes in order to terminate CB_1_ signaling, and by different classes of enzymes aiming transformation of eCBs into other classes of lipidergic signaling molecules, such as prostamides (Urquhart et al., 2015). The fact that eCBs belong to the family of polyunsaturated fatty acids makes them indeed attractive substrates for enzymatic oxidation, as induced by lipoxygenases (LOX), cyclooxygenases (COX), or cytochrome P450 (Rouzer and Marnett, 2011). Numerous eCBs have been described so far and in addition to 2-AG it is arachidonoylethanolamine (AEA) representing by far the best-studied intrinsic ligand of CB_1_ today. However, beside CB_1_ and CB_2_ as the most relevant G protein-coupled receptors of cannabinoids, it is likely that eCBs also act upon several other G protein-coupled receptors, such as GPR18, GPR55, and GPR119. These former orphan receptors are putative candidates for nomination of CB_3_, however their relevance in feeding regulation has to be further investigated. Nevertheless, it appeared that GPR18 and GPR55 signaling is involved in processes of metabolic dysfunction (Liu et al., 2015; Rajaraman et al., 2016). Besides G protein-coupled receptors, eCBs such as AEA were also shown to act upon other types of receptors, such as transient receptor potential (TRP) vanilloid 1 (Pertwee, 2010). Moreover, several enzymes involved in eCB biosynthesis, such as the AEA synthesizing N-acyl phosphatidylethanolamine-specific phospholipase D (NAPE-PLD) not only give rise to the CB_1_ ligand AEA, but also to structural very similar lipid messengers that do not bind and activate CB_1_. In this, it was shown that oleoylethanolamine (OEA) and palmitoylethanolamine (PEA), as close related lipids of AEA, bind to PPARs (Fu et al., 2003; Lo Verme et al., 2005; Gaetani et al., 2010), which are well-known to contribute in control of glucose, lipid, and energy metabolism (Grygiel-Gorniak, 2014). Thus, the overall metabolic role of the enzymes in the ECS, beside CB_1_, may deliver future targets for therapeutic interventions in control of feeding behavior. Indeed, targeted lipidomics of different brain regions derived from mice either deficient for CB_1_, the AEA degrading enzyme FAAH or the aforementioned 2-AG degrading MAGL revealed that AEA and 2-AG hydrolyzing enzymes, when compared to CB_1_, link the ECS to a broader lipid signaling network in contrasting ways, which again may open an avenue in altering neurotransmission and behaviors independently of CB_1_ signaling (Leishman et al., 2016a). This assumption is further supported by another lipidomic analysis. In this, mice deficient for NAPE-PLD not only displayed a shift in the concentration of AEA, but also shifted several other lipids, not binding to CB_1_, such as OEA and PEA, that as mentioned before signal upon different metabolic relevant targets, such as PPARs (Leishman et al., 2016b).

## Outlook

Actually, there has been significant increase of knowledge about central CB_1_ signaling in control of feeding behavior. Despite the significant setback that occurred in the past on clinical use of CB_1_ inverse agonists in order to treat overeating, there still is strong confidence in the field that the recent discoveries on central CB_1_ signaling soon will leverage the therapeutic potential of CB_1_.

## Author contributions

MK designed this review, including Figure 1.

### Conflict of interest statement

The author declares that the research was conducted in the absence of any commercial or financial relationships that could be construed as a potential conflict of interest.

## References

[B1] Aigner M. Treasure J. Kaye W. Kasper S. Disorders W.T.F.O.E. (2011). World Federation of Societies of Biological Psychiatry (WFSBP) guidelines for the pharmacological treatment of eating disorders. World J. Biol. Psychiatry 12, 400–443. 10.3109/15622975.2011.602720 21961502

[B2] Alonso-Alonso M. Woods S. C. Pelchat M. Grigson P. S. Stice E. Farooqi S.. (2015). Food reward system: current perspectives and future research needs. Nutr. Rev. 73, 296–307. 10.1093/nutrit/nuv002 26011903 PMC4477694

[B3] Alpar A. Harkany T. (2013). Orexin neurons use endocannabinoids to break obesity-induced inhibition. Proc. Natl. Acad. Sci. U.S.A. 110, 9625–9626. 10.1073/pnas.1307389110 23720305 PMC3683706

[B4] Argente-Arizon P. Guerra-Cantera S. Garcia-Segura L. M. Argente J. Chowen J. A. (2017). Glial cells and energy balance. J. Mol. Endocrinol. 58, R59–R71. 10.1530/JME-16-0182 27864453

[B5] Baufeld C. Osterloh A. Prokop S. Miller K. R. Heppner F. L. (2016). High-fat diet-induced brain region-specific phenotypic spectrum of CNS resident microglia. Acta Neuropathol. 132, 361–375. 10.1007/s00401-016-1595-4 27393312 PMC4992033

[B6] Bellocchio L. Lafenetre P. Cannich A. Cota D. Puente N. Grandes P.. (2010). Bimodal control of stimulated food intake by the endocannabinoid system. Nat. Neurosci. 13, 281–283. 10.1038/nn.2494 20139974

[B7] Benard G. Massa F. Puente N. Lourenco J. Bellocchio L. Soria-Gomez E.. (2012). Mitochondrial CB(1) receptors regulate neuronal energy metabolism. Nat. Neurosci. 15, 558–564. 10.1038/nn.3053 22388959

[B8] Bosier B. Bellocchio L. Metna-Laurent M. Soria-Gomez E. Matias I. Hebert-Chatelain E.. (2013). Astroglial CB1 cannabinoid receptors regulate leptin signaling in mouse brain astrocytes. Mol. Metab. 2, 393–404. 10.1016/j.molmet.2013.08.001 24327955 PMC3854987

[B9] Busquets Garcia A. Soria-Gomez E. Bellocchio L. Marsicano G. (2016). Cannabinoid receptor type-1: breaking the dogmas. F1000Res 2016:5 10.12688/f1000research.8245.1 PMC4879932 27239293

[B10] Campbell J. N. Macosko E. Z. Fenselau H. Pers T. H. Lyubetskaya A. Tenen D.. (2017). A molecular census of arcuate hypothalamus and median eminence cell types. Nat. Neurosci. 20, 484–496. 10.1038/nn.4495 28166221 PMC5323293

[B11] Cardinal P. Bellocchio L. Clark S. Cannich A. Klugmann M. Lutz B.. (2012). Hypothalamic CB1 cannabinoid receptors regulate energy balance in mice. Endocrinology 153, 4136–4143. 10.1210/en.2012-1405 22778221

[B12] Castillo P. E. Younts T. J. Chavez A. E. Hashimotodani Y. (2012). Endocannabinoid signaling and synaptic function. Neuron 76, 70–81. 10.1016/j.neuron.2012.09.020 23040807 PMC3517813

[B13] Chen Y. Lin Y. C. Kuo T. W. Knight Z. A. (2015). Sensory detection of food rapidly modulates arcuate feeding circuits. Cell 160, 829–841. 10.1016/j.cell.2015.01.033 25703096 PMC4373539

[B14] Colombo G. Agabio R. Diaz G. Lobina C. Reali R. Gessa G. L. (1998). Appetite suppression and weight loss after the cannabinoid antagonist SR 141716. Life Sci. 63, PL113–PL117. 10.1016/S0024-3205(98)00322-1 9718088

[B15] Cota D. Marsicano G. Tschop M. Grubler Y. Flachskamm C. Schubert M.. (2003). The endogenous cannabinoid system affects energy balance via central orexigenic drive and peripheral lipogenesis. J. Clin. Invest. 112, 423–431. 10.1172/JCI17725 12897210 PMC166293

[B16] Cristino L. Busetto G. Imperatore R. Ferrandino I. Palomba L. Silvestri C.. (2013). Obesity-driven synaptic remodeling affects endocannabinoid control of orexinergic neurons. Proc. Natl. Acad. Sci. U.S.A. 110, E2229–E2238. 10.1073/pnas.1219485110 23630288 PMC3683753

[B17] Devane W. A. Hanus L. Breuer A. Pertwee R. G. Stevenson L. A. Griffin G.. (1992). Isolation and structure of a brain constituent that binds to the cannabinoid receptor. Science 258, 1946–1949. 10.1126/science.1470919 1470919

[B18] Di Marzo V. Goparaju S. K. Wang L. Liu J. Batkai S. Jarai Z.. (2001). Leptin-regulated endocannabinoids are involved in maintaining food intake. Nature 410, 822–825. 10.1038/35071088 11298451

[B19] Di Marzo V. Ligresti A. Cristino L. (2009). The endocannabinoid system as a link between homoeostatic and hedonic pathways involved in energy balance regulation. Int. J. Obes. 33(Suppl. 2), S18–S24. 10.1038/ijo.2009.67 19528974

[B20] Dietrich M. O. Horvath T. L. (2012). Limitations in anti-obesity drug development: the critical role of hunger-promoting neurons. Nat. Rev. Drug Discov. 11, 675–691. 10.1038/nrd3739 22858652

[B21] Dodd G. T. Mancini G. Lutz B. Luckman S. M. (2010). The peptide hemopressin acts through CB1 cannabinoid receptors to reduce food intake in rats and mice. J. Neurosci. 30, 7369–7376. 10.1523/JNEUROSCI.5455-09.2010 20505104 PMC6632410

[B22] Dodd G. T. Worth A. A. Hodkinson D. J. Srivastava R. K. Lutz B. Williams S. R.. (2013). Central functional response to the novel peptide cannabinoid, hemopressin. Neuropharmacology 71, 27–36. 10.1016/j.neuropharm.2013.03.007 23542442

[B23] Engeli S. (2008). Dysregulation of the endocannabinoid system in obesity. J. Neuroendocrinol. 20(Suppl. 1), 110–115. 10.1111/j.1365-2826.2008.01683.x 18426509

[B24] Fenselau H. Campbell J. N. Verstegen A. M. Madara J. C. Xu J. Shah B. P.. (2017). A rapidly acting glutamatergic ARC–>PVH satiety circuit postsynaptically regulated by alpha-MSH. Nat. Neurosci. 20, 42–51. 10.1038/nn.4442 27869800 PMC5191921

[B25] Fisette A. Tobin S. Decarie-Spain L. Bouyakdan K. Peyot M. L. Madiraju S. R.. (2016). Alpha/beta-hydrolase domain 6 in the ventromedial hypothalamus controls energy metabolism flexibility. Cell Rep. 17, 1217–1226. 10.1016/j.celrep.2016.10.004 27783937

[B26] Fu J. Gaetani S. Oveisi F. Lo Verme J. Serrano A. Rodriguez De Fonseca F.. (2003). Oleylethanolamide regulates feeding and body weight through activation of the nuclear receptor PPAR-alpha. Nature 425, 90–93. 10.1038/nature01921 12955147

[B27] Gaetani S. Fu J. Cassano T. Dipasquale P. Romano A. Righetti L.. (2010). The fat-induced satiety factor oleoylethanolamide suppresses feeding through central release of oxytocin. J. Neurosci. 30, 8096–8101. 10.1523/JNEUROSCI.0036-10.2010 20554860 PMC2900249

[B28] Gao X. B. Horvath T. L. (2016). Feeding behavior: hypocretin/orexin neurons act between food seeking and eating. Curr. Biol. 26, R845–R847. 10.1016/j.cub.2016.07.069 27676302

[B29] Grygiel-Gorniak B. (2014). Peroxisome proliferator-activated receptors and their ligands: nutritional and clinical implications–a review. Nutr. J. 13:17. 10.1186/1475-2891-13-17 24524207 PMC3943808

[B30] Han J. Kesner P. Metna-Laurent M. Duan T. Xu L. Georges F.. (2012). Acute cannabinoids impair working memory through astroglial CB1 receptor modulation of hippocampal LTD. Cell 148, 1039–1050. 10.1016/j.cell.2012.01.037 22385967

[B31] Harkany T. Horvath T. L. (2017). (S)Pot on mitochondria: cannabinoids disrupt cellular respiration to limit neuronal activity. Cell Metab. 25, 8–10. 10.1016/j.cmet.2016.12.020 28076767

[B32] Hebert-Chatelain E. Desprez T. Serrat R. Bellocchio L. Soria-Gomez E. Busquets-Garcia A.. (2016). A cannabinoid link between mitochondria and memory. Nature 539, 555–559. 10.1038/nature20127 27828947

[B33] Hebert-Chatelain E. Reguero L. Puente N. Lutz B. Chaouloff F. Rossignol R.. (2014). Cannabinoid control of brain bioenergetics: exploring the subcellular localization of the CB1 receptor. Mol. Metab. 3, 495–504. 10.1016/j.molmet.2014.03.007 24944910 PMC4060213

[B34] Heimann A. S. Gomes I. Dale C. S. Pagano R. L. Gupta A. De Souza L. L.. (2007). Hemopressin is an inverse agonist of CB1 cannabinoid receptors. Proc. Natl. Acad. Sci. U.S.A. 104, 20588–20593. 10.1073/pnas.0706980105 18077343 PMC2154475

[B35] Hentges S. T. Low M. J. Williams J. T. (2005). Differential regulation of synaptic inputs by constitutively released endocannabinoids and exogenous cannabinoids. J. Neurosci. 25, 9746–9751. 10.1523/JNEUROSCI.2769-05.2005 16237178 PMC6725733

[B36] Herkenham M. Lynn A. B. Little M. D. Johnson M. R. Melvin L. S. De Costa B. R.. (1990). Cannabinoid receptor localization in brain. Proc. Natl. Acad. Sci. U.S.A. 87, 1932–1936. 10.1073/pnas.87.5.1932 2308954 PMC53598

[B37] Horvath T. L. (2003). Endocannabinoids and the regulation of body fat: the smoke is clearing. J. Clin. Invest. 112, 323–326. 10.1172/JCI19376 12897199 PMC166302

[B38] Hua T. Vemuri K. Pu M. Qu L. Han G. W. Wu Y.. (2016). Crystal structure of the human cannabinoid receptor CB1. Cell 167, 750 e714–762 e714. 10.1016/j.cell.2016.10.004 27768894 PMC5322940

[B39] Jamshidi N. Taylor D. A. (2001). Anandamide administration into the ventromedial hypothalamus stimulates appetite in rats. Br. J. Pharmacol. 134, 1151–1154. 10.1038/sj.bjp.0704379 11704633 PMC1573067

[B40] Kim J. G. Suyama S. Koch M. Jin S. Argente-Arizon P. Argente J.. (2014). Leptin signaling in astrocytes regulates hypothalamic neuronal circuits and feeding. Nat. Neurosci. 17, 908–910. 10.1038/nn.3725 24880214 PMC4113214

[B41] Koch M. Horvath T. L. (2014). Molecular and cellular regulation of hypothalamic melanocortin neurons controlling food intake and energy metabolism. Mol. Psychiatry 19, 752–761. 10.1038/mp.2014.30 24732669

[B42] Koch M. Varela L. Kim J. G. Kim J. D. Hernandez-Nuno F. Simonds S. E.. (2015). Hypothalamic POMC neurons promote cannabinoid-induced feeding. Nature 519, 45–50. 10.1038/nature14260 25707796 PMC4496586

[B43] Kola B. Farkas I. Christ-Crain M. Wittmann G. Lolli F. Amin F.. (2008). The orexigenic effect of ghrelin is mediated through central activation of the endogenous cannabinoid system. PLoS ONE 3:e1797. 10.1371/journal.pone.0001797 18335063 PMC2258435

[B44] Kruger R. P. (2016). Harvesting benefits from cannabinoids. Cell 167, 1663–1665. 10.1016/j.cell.2016.12.001 27984713

[B45] Leishman E. Cornett B. Spork K. Straiker A. Mackie K. Bradshaw H. B. (2016a). Broad impact of deleting endogenous cannabinoid hydrolyzing enzymes and the CB1 cannabinoid receptor on the endogenous cannabinoid-related lipidome in eight regions of the mouse brain. Pharmacol. Res. 110, 159–172. 10.1016/j.phrs.2016.04.020 27109320 PMC4914450

[B46] Leishman E. Mackie K. Luquet S. Bradshaw H. B. (2016b). Lipidomics profile of a NAPE-PLD KO mouse provides evidence of a broader role of this enzyme in lipid metabolism in the brain. Biochim. Biophys. Acta 1861, 491–500. 10.1016/j.bbalip.2016.03.003 26956082 PMC4909477

[B47] Liu B. Song S. Jones P. M. Persaud S. J. (2015). GPR55: from orphan to metabolic regulator? Pharmacol. Ther. 145, 35–42. 10.1016/j.pharmthera.2014.06.007 24972076

[B48] Lo Verme J. Fu J. Astarita G. La Rana G. Russo R. Calignano A.. (2005). The nuclear receptor peroxisome proliferator-activated receptor-alpha mediates the anti-inflammatory actions of palmitoylethanolamide. Mol. Pharmacol. 67, 15–19. 10.1124/mol.104.006353 15465922

[B49] Lutz B. Marsicano G. Maldonado R. Hillard C. J. (2015). The endocannabinoid system in guarding against fear, anxiety and stress. Nat. Rev. Neurosci. 16, 705–718. 10.1038/nrn4036 26585799 PMC5871913

[B50] Matsuda L. A. Lolait S. J. Brownstein M. J. Young A. C. Bonner T. I. (1990). Structure of a cannabinoid receptor and functional expression of the cloned cDNA. Nature 346, 561–564. 10.1038/346561a0 2165569

[B51] Mazier W. Saucisse N. Gatta-Cherifi B. Cota D. (2015). The endocannabinoid system: pivotal orchestrator of obesity and metabolic disease. Trends Endocrinol. Metab. 26, 524–537. 10.1016/j.tem.2015.07.007 26412154

[B52] Melis T. Succu S. Sanna F. Boi A. Argiolas A. Melis M. R. (2007). The cannabinoid antagonist SR 141716A (Rimonabant) reduces the increase of extra-cellular dopamine release in the rat nucleus accumbens induced by a novel high palatable food. Neurosci. Lett. 419, 231–235. 10.1016/j.neulet.2007.04.012 17462824

[B53] Metna-Laurent M. Marsicano G. (2015). Rising stars: modulation of brain functions by astroglial type-1 cannabinoid receptors. Glia 63, 353–364. 10.1002/glia.22773 25452006

[B54] Morello G. Imperatore R. Palomba L. Finelli C. Labruna G. Pasanisi F.. (2016). Orexin-A represses satiety-inducing POMC neurons and contributes to obesity via stimulation of endocannabinoid signaling. Proc. Natl. Acad. Sci. U.S.A. 113, 4759–4764. 10.1073/pnas.1521304113 27071101 PMC4855580

[B55] Morozov Y. M. Koch M. Rakic P. Horvath T. L. (2017). Cannabinoid type 1 receptor-containing axons innervate NPY/AgRP neurons in the mouse arcuate nucleus. Mol. Metab. 6, 374–381. 10.1016/j.molmet.2017.01.004 28377876 PMC5369208

[B56] Muller T. D. Nogueiras R. Andermann M. L. Andrews Z. B. Anker S. D. Argente J.. (2015). Ghrelin. Mol. Metab. 4, 437–460. 10.1016/j.molmet.2015.03.005 26042199 PMC4443295

[B57] Munzberg H. Qualls-Creekmore E. Yu S. Morrison C. D. Berthoud H. R. (2016). Hedonics act in unison with the homeostatic system to unconsciously control body weight. Front Nutr 3:6. 10.3389/fnut.2016.00006 26913284 PMC4753312

[B58] Navarrete M. Araque A. (2010). Endocannabinoids potentiate synaptic transmission through stimulation of astrocytes. Neuron 68, 113–126. 10.1016/j.neuron.2010.08.043 20920795

[B59] Nogueiras R. Veyrat-Durebex C. Suchanek P. M. Klein M. Tschop J. Caldwell C.. (2008). Peripheral, but not central, CB1 antagonism provides food intake-independent metabolic benefits in diet-induced obese rats. Diabetes 57, 2977–2991. 10.2337/db08-0161 18716045 PMC2570394

[B60] Oliveira Da Cruz J. F. Robin L. M. Drago F. Marsicano G. Metna-Laurent M. (2016). Astroglial type-1 cannabinoid receptor (CB1): a new player in the tripartite synapse. Neuroscience 323, 35–42. 10.1016/j.neuroscience.2015.05.002 25967266

[B61] Palomba L. Silvestri C. Imperatore R. Morello G. Piscitelli F. Martella A.. (2015). Negative regulation of leptin-induced Reactive Oxygen Species (ROS) formation by cannabinoid CB1 receptor activation in hypothalamic neurons. J. Biol. Chem. 290, 13669–13677. 10.1074/jbc.M115.646885 25869131 PMC4447947

[B62] Pandurangan M. Hwang I. (2015). Systemic mechanism of taste, flavour and palatability in brain. Appl. Biochem. Biotechnol. 175, 3133–3147. 10.1007/s12010-015-1488-3 25733187

[B63] Park R. J. Godier L. R. Cowdrey F. A. (2014). Hungry for reward: how can neuroscience inform the development of treatment for Anorexia Nervosa? Behav. Res. Ther. 62, 47–59. 10.1016/j.brat.2014.07.007 25151600

[B64] Patel S. Cone R. D. (2015). Neuroscience: a cellular basis for the munchies. Nature 519, 38–40. 10.1038/nature14206 25707800

[B65] Pertwee R. G. (2010). Receptors and channels targeted by synthetic cannabinoid receptor agonists and antagonists. Curr. Med. Chem. 17, 1360–1381. 10.2174/092986710790980050 20166927 PMC3013229

[B66] Pertwee R. G. (2014). Elevating endocannabinoid levels: pharmacological strategies and potential therapeutic applications. Proc. Nutr. Soc. 73, 96–105. 10.1017/S0029665113003649 24135210

[B67] Pinto S. Roseberry A. G. Liu H. Diano S. Shanabrough M. Cai X.. (2004). Rapid rewiring of arcuate nucleus feeding circuits by leptin. Science 304, 110–115. 10.1126/science.1089459 15064421

[B68] Piomelli D. (2003). The molecular logic of endocannabinoid signalling. Nat. Rev. Neurosci. 4, 873–884. 10.1038/nrn1247 14595399

[B69] Rajaraman G. Simcocks A. Hryciw D. H. Hutchinson D. S. Mcainch A. J. (2016). G protein coupled receptor 18: a potential role for endocannabinoid signaling in metabolic dysfunction. Mol. Nutr. Food Res. 60, 92–102. 10.1002/mnfr.201500449 26337420

[B70] Reuter S. E. Martin J. H. (2016). Pharmacokinetics of cannabis in cancer cachexia-anorexia syndrome. Clin. Pharmacokinet. 55, 807–812. 10.1007/s40262-015-0363-2 26883879

[B71] Romanov R. A. Zeisel A. Bakker J. Girach F. Hellysaz A. Tomer R.. (2017). Molecular interrogation of hypothalamic organization reveals distinct dopamine neuronal subtypes. Nat. Neurosci. 20, 176–188. 10.1038/nn.4462 27991900 PMC7615022

[B72] Rouzer C. A. Marnett L. J. (2011). Endocannabinoid oxygenation by cyclooxygenases, lipoxygenases, and cytochromes P450: cross-talk between the eicosanoid and endocannabinoid signaling pathways. Chem. Rev. 111, 5899–5921. 10.1021/cr2002799 21923193 PMC3191732

[B73] Savinainen J. R. Saario S. M. Laitinen J. T. (2012). The serine hydrolases MAGL, ABHD6 and ABHD12 as guardians of 2-arachidonoylglycerol signalling through cannabinoid receptors. Acta Physiol. 204, 267–276. 10.1111/j.1748-1716.2011.02280.x 21418147 PMC3320662

[B74] Scarlett J. M. Marks D. L. (2005). The use of melanocortin antagonists in cachexia of chronic disease. Expert Opin. Investig. Drugs 14, 1233–1239. 10.1517/13543784.14.10.1233 16185165

[B75] Seeley R. J. Berridge K. C. (2015). The hunger games. Cell 160, 805–806. 10.1016/j.cell.2015.02.028 25723156

[B76] Shao Z. Yin J. Chapman K. Grzemska M. Clark L. Wang J. (2016). High-resolution crystal structure of the human CB1 cannabinoid receptor. Nature 540, 602–606. 10.1038/nature20613 PMC5433929 27851727

[B77] Silvestri C. Di Marzo V. (2013). The endocannabinoid system in energy homeostasis and the etiopathology of metabolic disorders. Cell Metab. 17, 475–490. 10.1016/j.cmet.2013.03.001 23562074

[B78] Simiand J. Keane M. Keane P. E. Soubrie P. (1998). SR 141716, a CB1 cannabinoid receptor antagonist, selectively reduces sweet food intake in marmoset. Behav. Pharmacol. 9, 179–181. 10065938

[B79] Soria-Gomez E. Bellocchio L. Reguero L. Lepousez G. Martin C. Bendahmane M.. (2014a). The endocannabinoid system controls food intake via olfactory processes. Nat. Neurosci. 17, 407–415. 10.1038/nn.3647 24509429

[B80] Soria-Gomez E. Massa F. Bellocchio L. Rueda-Orozco P. E. Ciofi P. Cota D. (2014b). Cannabinoid type-1 receptors in the paraventricular nucleus of the hypothalamus inhibit stimulated food intake. Neuroscience 263C, 46–53. 10.1016/j.neuroscience.2014.01.005 24434770

[B81] Tam J. Cinar R. Liu J. Godlewski G. Wesley D. Jourdan T.. (2012). Peripheral cannabinoid-1 receptor inverse agonism reduces obesity by reversing leptin resistance. Cell Metab. 16, 167–179. 10.1016/j.cmet.2012.07.002 22841573 PMC3832894

[B82] Thibault K. Carrel D. Bonnard D. Gallatz K. Simon A. Biard M.. (2013). Activation-dependent subcellular distribution patterns of CB1 cannabinoid receptors in the rat forebrain. Cereb. Cortex 23, 2581–2591. 10.1093/cercor/bhs240 22892424

[B83] Urquhart P. Nicolaou A. Woodward D. F. (2015). Endocannabinoids and their oxygenation by cyclo-oxygenases, lipoxygenases and other oxygenases. Biochim. Biophys. Acta 1851, 366–376. 10.1016/j.bbalip.2014.12.015 25543004

[B84] Vallee M. Vitiello S. Bellocchio L. Hebert-Chatelain E. Monlezun S. Martin-Garcia E.. (2014). Pregnenolone can protect the brain from cannabis intoxication. Science 343, 94–98. 10.1126/science.1243985 24385629 PMC4057431

[B85] Varela L. Horvath T. L. (2012). Leptin and insulin pathways in POMC and AgRP neurons that modulate energy balance and glucose homeostasis. EMBO Rep. 13, 1079–1086. 10.1038/embor.2012.174 23146889 PMC3512417

[B86] Verty A. N. Boon W. M. Mallet P. E. Mcgregor I. S. Oldfield B. J. (2009). Involvement of hypothalamic peptides in the anorectic action of the CB receptor antagonist rimonabant (SR 141716). Eur. J. Neurosci. 29, 2207–2216. 10.1111/j.1460-9568.2009.06750.x 19490094

[B87] Vogt M. C. Bruning J. C. (2013). CNS insulin signaling in the control of energy homeostasis and glucose metabolism - from embryo to old age. Trends Endocrinol. Metab. 24, 76–84. 10.1016/j.tem.2012.11.004 23265947

[B88] Volkow N. D. Wang G. J. Baler R. D. (2011). Reward, dopamine and the control of food intake: implications for obesity. Trends Cogn. Sci. 15, 37–46. 10.1016/j.tics.2010.11.001 21109477 PMC3124340

[B89] Walter L. Stella N. (2004). Cannabinoids and neuroinflammation. Br. J. Pharmacol. 141, 775–785. 10.1038/sj.bjp.0705667 14757702 PMC1574256

[B90] Whiting P. F. Wolff R. F. Deshpande S. Di Nisio M. Duffy S. Hernandez A. V.. (2015). Cannabinoids for medical use: a systematic review and meta-analysis. JAMA 313, 2456–2473. 10.1001/jama.2015.6358 26103030

[B91] Williams C. M. Kirkham T. C. (1999). Anandamide induces overeating: mediation by central cannabinoid (CB1) receptors. Psychopharmacology 143, 315–317. 10.1007/s002130050953 10353436

[B92] Zhang X. van den Pol A. N. (2016). Hypothalamic arcuate nucleus tyrosine hydroxylase neurons play orexigenic role in energy homeostasis. Nat. Neurosci. 19, 1341–1347. 10.1038/nn.4372 27548245 PMC6402046

